# Modification of Hemodynamic and Immune Responses to Exposure with a Weak Antigen by the Expression of a Hypomorphic BMPR2 Gene

**DOI:** 10.1371/journal.pone.0055180

**Published:** 2013-01-29

**Authors:** Sung-Hyun Park, Wen-Chi Chen, Carol Hoffman, Leigh M. Marsh, James West, Gabriele Grunig

**Affiliations:** 1 Department of Environmental Medicine, New York University School of Medicine, Tuxedo, New York, United States of America; 2 Ludwig Boltzmann Institute for Lung Vascular Research, Graz, Austria; 3 Division of Allergy, Pulmonary, and Critical Care Medicine, Department of Medicine, Vanderbilt University Medical Center, Nashville, Tennessee, United States of America; 4 Division of Pulmonary Medicine, Department of Medicine, New York University School of Medicine, New York, New York, United States of America; University Hospital Freiburg, Germany

## Abstract

**Background:**

Hypomorphic mutations in the bone morphogenic protein receptor (BMPR2) confer a much greater risk for developing pulmonary arterial hypertension (PAH). However, not all carriers of a mutation in the BMPR2 gene suffer from PAH. We have previously shown that prolonged T helper 2 (Th2) responses in the lungs to a mild antigen delivered via the airways induce severe pulmonary arterial remodeling, but no pulmonary hypertension. The current studies were designed to test the idea that Th2 responses to a mild antigen together with the expression of a hypomorphic BMPR2 gene would trigger pulmonary hypertension.

**Methodology/Principal Findings:**

Mice that expressed a hypomorphic BMPR2 transgene (transgene-positive) and transgene-negative mice were either exposed to saline, or primed and exposed to a mild antigen (Ovalbumin) over a prolonged period of time. Only transgene-positive but not transgene-negative mice exposed to antigen developed significantly increased right ventricular systolic pressures, while both groups showed pulmonary artery remodeling with severe muscularization and airway inflammation to a similar degree. Antigen exposure resulted in a smaller increase in the percentage of Interleukin (IL)-13 positive T cells in the lymph nodes, and in a smaller increase in resistin-like-molecule (RELM)α expression and a decreased ratio of expression of IL-33 relative to its receptor (IL-1-receptor-like 1, IL1RL1-ST2) in the right ventricles of transgene-positive mice compared to transgene-negative animals. Furthermore, only antigen-challenged transgene-positive mice showed a significant increase in Interferon (IFN)γ positive T cells over saline-exposed controls.

**Conclusions/Significance:**

Our study suggests that exposure with a mild Th2 antigen can trigger pulmonary hypertension on the background of the expression of a hypomorphic BMPR2 gene and that conversely, the expression of the hypomorphic BMPR2 gene can alter the immune response to a mild, inhaled antigen.

## Introduction

Pulmonary arterial hypertension (PAH) is characterized by increased right ventricular systolic pressures and severe thickening of arteries due to smooth muscle cell hypertrophy and impaired ability to terminate repair responses [1], [2]. Our group has reported that severe pulmonary arterial remodeling can be caused by a T helper 2 (Th2) immune response to inhaled antigen [3].

Bone morphogenic protein receptor (BMPR) 2 is a member of the transforming growth factor (TGF) β superfamily of transmembrane serine/threonine kinases which binds bone morphogenetic proteins (BMP) [4]. BMPR2 signaling controls many developmental processes through multiple downstream pathways [5]–[7]. TGFβ and BMP regulate endothelial and smooth muscle cell proliferation and apoptosis with opposite and antagonistic effects [6], [8]. In humans, mutations in BMPR2 and the dysfunction of BMPR2 are a cause of hereditary PAH [9]–[12]. Reduced BMPR2 signaling disturbs the balance between BMPR2 and TGFβ signaling resulting in enhanced TGFβ signaling, and contributes to abnormal growth responses to BMPs and TGFβ [6], [8], [13]. Increased TGFβ signaling induces smooth muscle cell proliferation [9].

However, even though patients who are BMPR2 mutation carriers have a much greater chance of developing PAH, not all of them do [14], [15]. The variability in penetrance is thought to depend on genetic and environmental modifiers, including female gender [13], [16], [17]. Inflammatory responses are also thought to exacerbate the consequences of mutations in the BMPR2 gene [8], [18]–[21]. However, the exact mechanism by which inflammatory processes would initiate or exacerbate the PAH phenotype in BMPR2 mutation carriers remains to be elucidated.

Many mutations occur in the BMPR2 tail domain [15]. One such mutation is an arginine to nonsense mutation at amino acid 899 (R899X) found in family US33 [22]. Dr. West's group created transgenic mice that express the BMPR2 R899X mutation (BMPR2^R899X^) under the control of specific promoters to express the transgene in smooth muscle cells, or in all cells when induced by doxycycline [22]–[24]. Upon the expression of the hypomorphic BMPR2 transgene for several weeks, the mice developed elevated right ventricular systolic pressure and muscularization of pulmonary vessels. Using the BMPR2^R899X^ mouse, we tested the idea that the Th2 immune response to inhaled antigen would exacerbate the pulmonary hypertension phenotype in mice that express the hypomorphic BMPR2 transgene.

## Results

### Groups of animals studied

**Figure 1:**
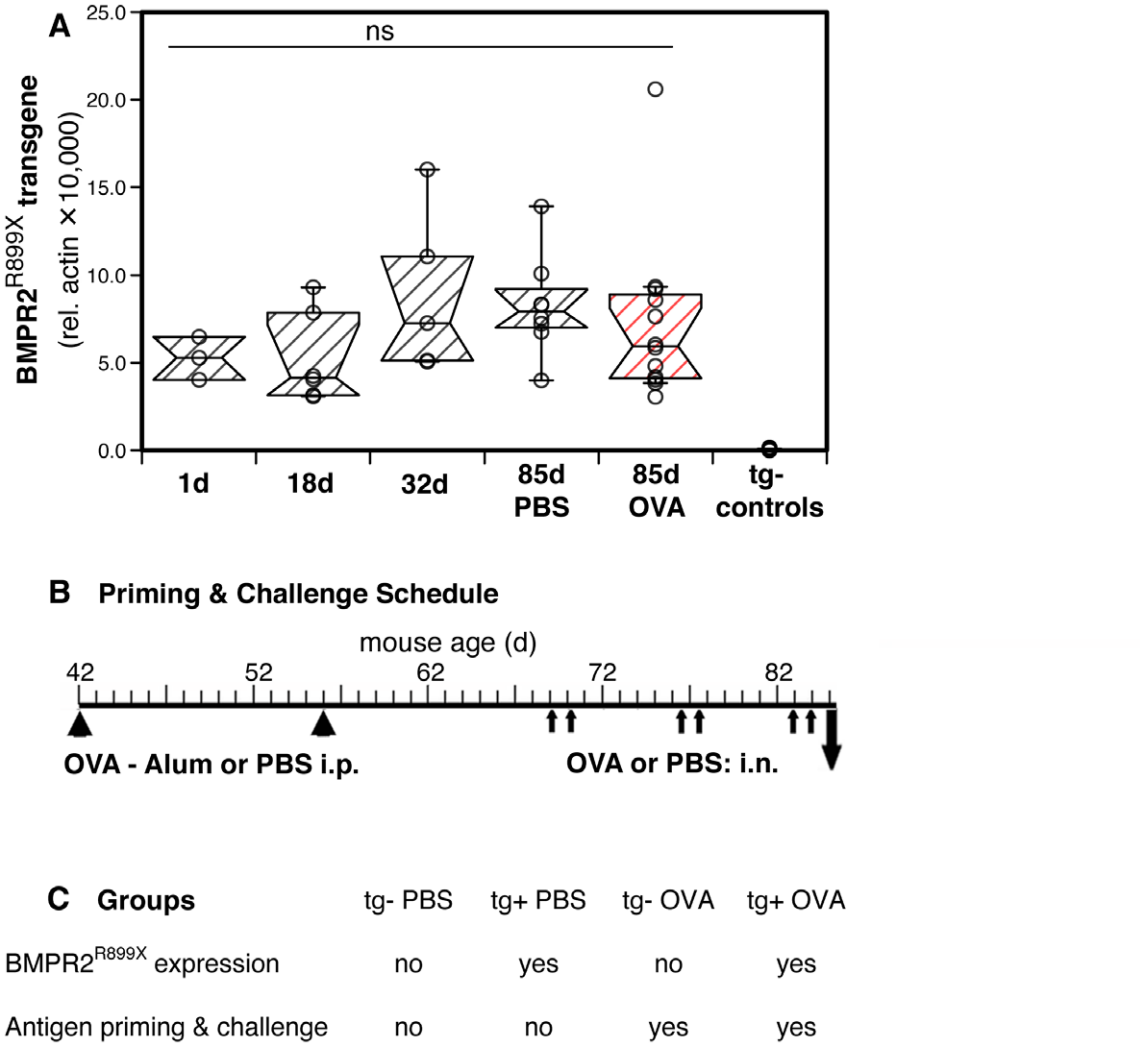
Expression of BMPR2^R899X^ transgene, experimental schedule and groups of animals studied. (**A**) Notched box and whisker plots extending to the 10^th^ and 90^th^ percentiles showing expression of mRNA for transgenic BMPR2^R899X^ (relative to β-actin; ×10,000) in heart tissues from animals aged 1 to 85 days old and transgene-negative (tg−) controls (n = 3–12). Data of individual mice are represented as dots. At 85 days old, mice were from the saline (PBS) or antigen (OVA) exposed groups as indicated. There were no statistically significant differences between pairs of groups (Mann Whitney U test, p>0.05). (**B**) Schematic representation of the experimental schedule for antigen (Ovalbumin, OVA) priming by intraperitoneal (i.p.) injections of OVA-Alum followed by intranasal (i.n.) challenge with soluble OVA. Control animals were given saline (PBS). (**C**) Study groups of animals as defined by BMPR2^R899X^ transgene expression and exposure to antigen or saline.

When housed in our mouse facility at the Department of Environmental Medicine, New York University, Tuxedo, NY, all BMPR2^R899X^ mice expressed the BMPR2^R899X^ transgene in the hearts (Fig. 1A) and lungs (data not shown). The expression of the BMPR2^R899X^ transgene was detected from the first day of birth and persisted at similar levels through the life of the animals (Fig. 1A). Expression levels in the heart were similar in the hearts of saline and antigen exposed animals (Fig. 1A) and independent of doxycycline administration in the food (data not shown). Four groups of animal were studied: mice that expressed the BMPR2^R899X^ transgene or were transgene-negative that were either given saline intranasally, or intraperitoneally primed and intranasally challenged with antigen (Fig. 1B, C).

### Right ventricular systolic pressure was increased in transgene-positive mice exposed to antigen

**Figure 2:**
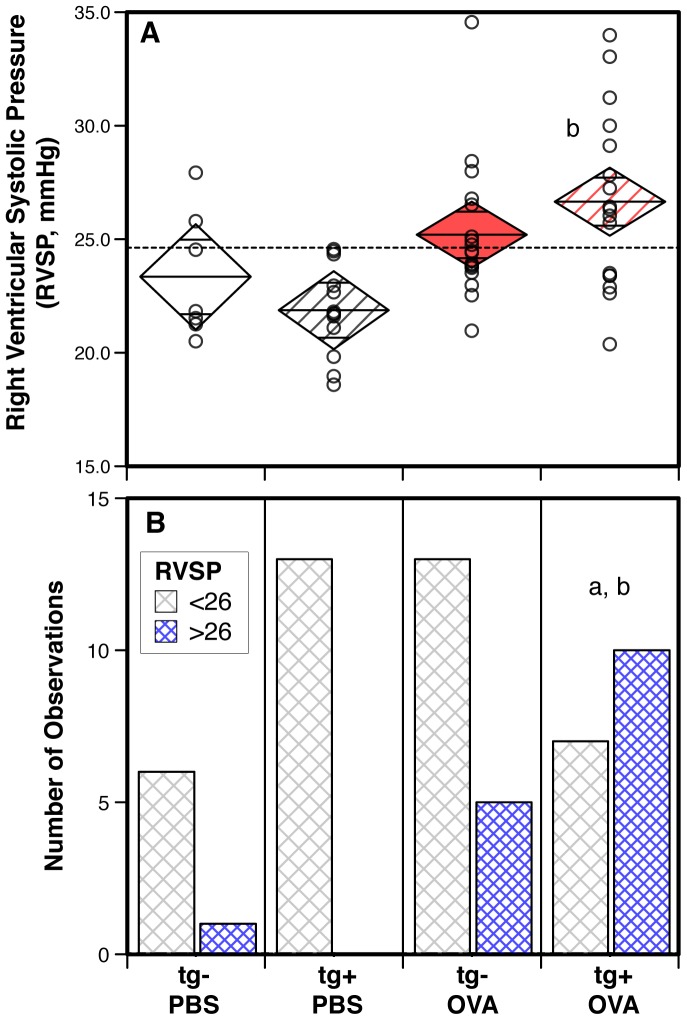
Increased right ventricular systolic pressures in mice that express the hypomorphic BMPR2 transgene and are exposed to antigen. (**A**) Diamond mean comparison plots show group means, upper and lower 95% confidence limits, overlap marks, for right ventricular systolic pressures (RVSP, mmHg) of saline (PBS) versus antigen (OVA) exposed mice stratified by transgene-negative (tg−) versus -positive (tg+). The dashed line represents the overall mean. Dots represent data from individual mice. Data were pooled from three independent experiments (n = 7 to 18). Pair-wise comparisons between groups were calculated with the Mann-Whitney U test (p<0.05; b: transgene-positive saline vs. antigen). (**B**) Bar graphs show the frequency of RVSP measurements that were lower or higher than 26 mmHg for each of the groups of animals. Chi-Square analysis of the data distribution between all groups was p = 0.0039; pair-wise comparisons calculated p<0.05 for a: transgene-positive antigen (OVA) vs. transgene negative saline (PBS); b: transgene-positive antigen vs. transgene-positive saline).

Transgene-positive animals that were primed and challenged with antigen had significantly increased right ventricular systolic pressures compared to saline exposed transgene-positive or transgene-negative groups of mice (Fig. 2). In contrast, antigen-exposed transgene-negative animals showed a trend to increased right ventricular systolic pressures that failed to reach statistical significance (Fig. 2). The significant increase in right ventricular systolic pressures in antigen-exposed transgene-positive but not in antigen-exposed transgene-negative mice (when compared to the respective saline exposed controls) was seen using two different analysis methods: comparison of the means (Fig. 2A), or analysis of the frequency of animals being below or above a threshold cutoff value for right ventricular systolic pressures that was set using the data from the saline-exposed animals (Fig. 2B). Of note, in our mouse facility, transgene-positive animals given saline did not show an increase in right ventricular systolic pressures when compared to the transgene-negative saline exposed group (Fig. 2).

### Muscularization of small pulmonary blood vessels

**Figure 3:**
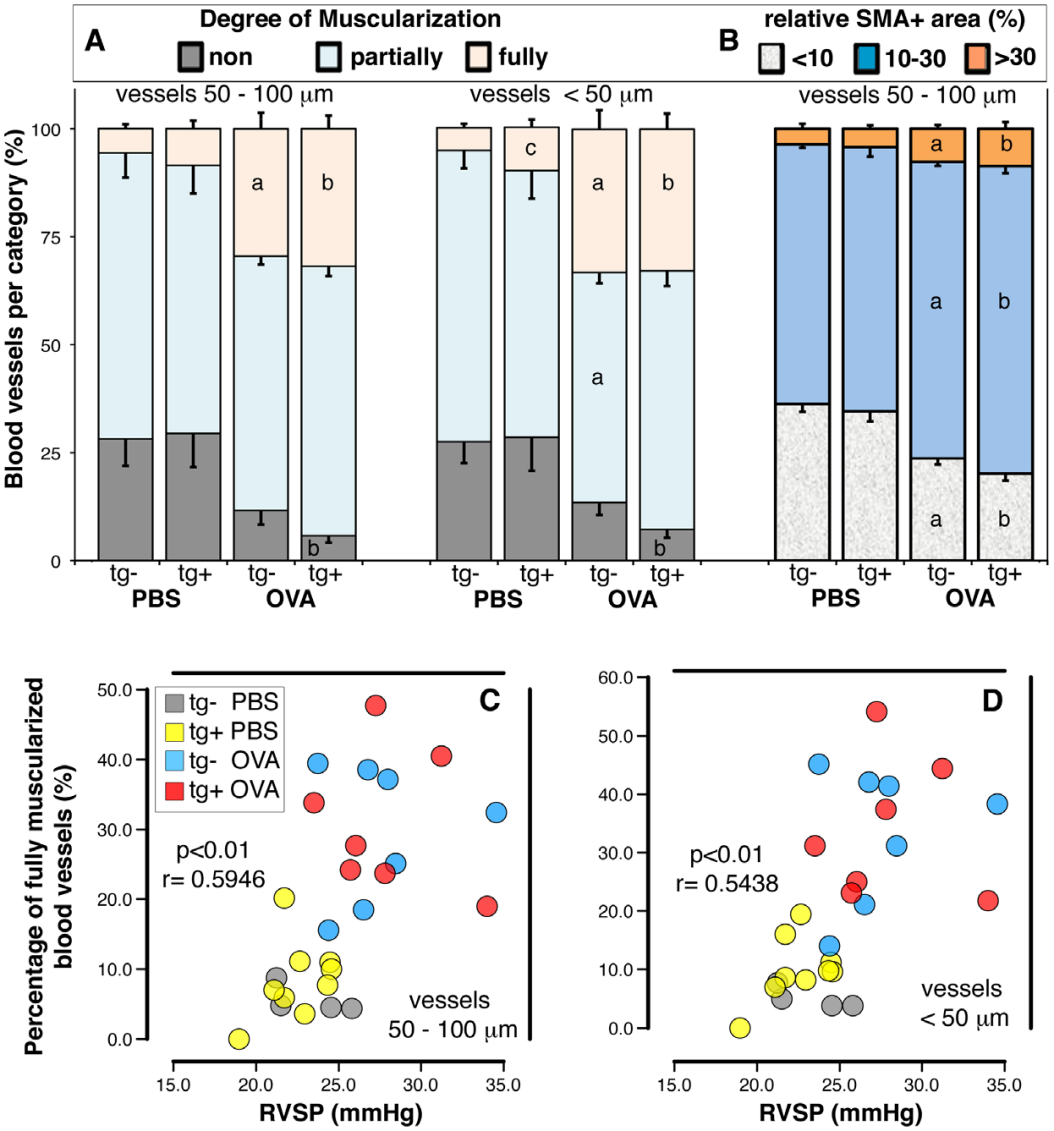
Muscularization of small blood vessels in the lungs: effects of the hypomorphic BMPR2 transgene and antigen exposure. **A**) Stacked bar graphs represent the percentage of non-muscularized, partially muscularized, and fully muscularized blood vessels that are 50–100, or <50 µm in diameter. **B**) Stacked bar graphs represent the percentage of bloods vessels (50–100 µm in diameter) for which the smooth muscle actin positive area was <10%, 10–30%, or >30% relative to the area of the total vessel. Data were pooled from two independent experiments (n = 4–9 per group with 60–1000, mean 400 vessels analyzed per mouse). Pair-wise comparisons were calculated with the t test with Welch's correction (p<0.05; a: transgene-negative saline (PBS) vs. antigen (OVA); b: transgene-positive saline vs. antigen; c: saline exposed transgene negative vs. transgene-positive). (**C, D**) Individual data points from two independent experiments (n = 4–7 per group) are shown. Each data point plots the percentage of fully muscularized blood vessels sized 50–100 µm (**C**) or <50 µm (**D**) in diameter against the right systolic ventricular pressure (RVSP, mmHg). Combined data from saline or antigen exposed groups of transgene-negative or trangene-positive mice (indicated by color code) were analyzed with the Spearman's Rank Order Correlation Coefficient test (p- and r- values are indicated).

Muscularization of pulmonary blood vessels was studied using a very sensitive, automated technique that is based on the detection of smooth muscle actin staining [25], [26]. Antigen exposure induced severe muscularization in small (50–100 µm in diameter) and very small (less than 50 µm in diameter) blood vessels (Fig. 3A, B). The percent of fully muscularized small and very small blood vessels was significantly correlated with right ventricular pressures (Fig. 3C, D). As previously reported by Dr. West's group [22], among the saline exposed groups of mice, transgene-positive mice had a higher percentage of fully muscularized very small blood vessels when compared to transgene-negative animals (Fig. 3A). There was no difference between groups of antigen exposed transgene-negative or transgene–positive animals with respect to the degree of muscularization or the relative smooth muscle actin positive area of the pulmonary blood vessels (Fig. 3A, B).

### Histological changes in the lungs of antigen challenged animals

**Figure 4:**
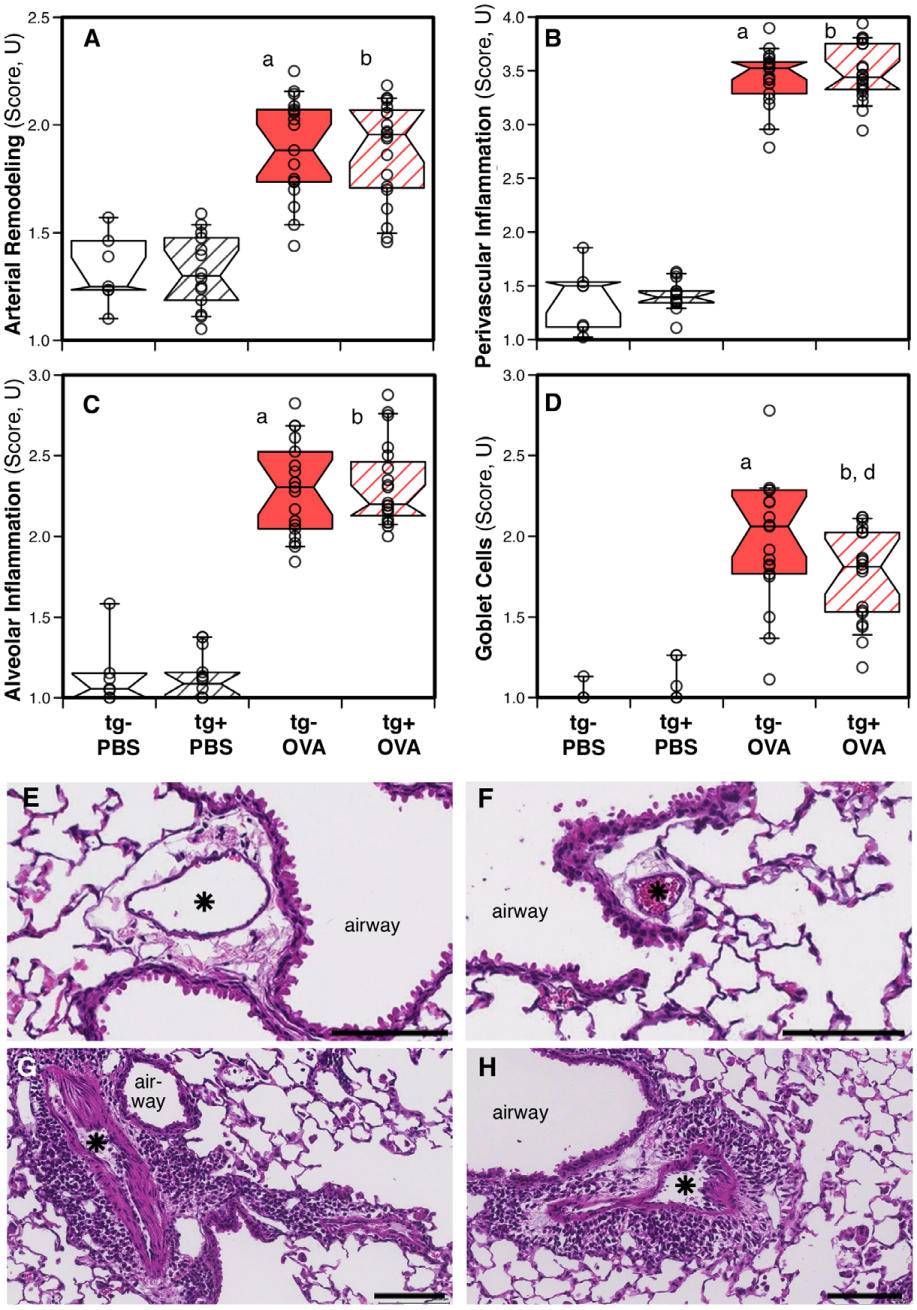
Histological changes in the lungs: effects of the hypomorphic BMPR2 transgene and antigen exposure. (**A–D**) Notched box and whisker plots extending to the 10^th^ and 90^th^ percentiles showing scores (1-normal to 4-severe change) for (**A**) pulmonary arterial remodeling, (**B**) perivascular inflammation, (**C**) alveolar inflammation, and (**D**) goblet cells. Data of individual mice are represented as dots. Data were pooled from three independent experiments (n = 7 to 20 per group). Pair-wise comparisons between groups were calculated with the Mann-Whitney U test (p<0.05; a: transgene-negative saline (PBS) vs. antigen (OVA); b: transgene-positive saline vs. antigen; d: antigen exposed transgene-negative vs. transgene-positive). (**E–H**) Scanned representative images show sections of lungs from saline, transgene-negative (**E**), saline, transgene-positive (**F**), antigen, transgene-negative (**G**), or antigen, transgene-positive (**H**) mice. The images of the antigen exposed mice show pulmonary arterial remodeling, perivascular inflammation and alveolar inflammation. For reference the airways (airway) and pulmonary arteries (*) are indicated; the scale bar represents 100 µm. Sections were stained with hematoxylin and eosin.

Lung histology was analyzed by scoring using previously published techniques [3], [27]–[30] from each of the animals included in this study (Fig. 4). Antigen-exposed transgene-positive or transgene–negative animals developed pulmonary arterial remodeling (Fig. 4A), perivascular inflammation (Fig. 4B), alveolar inflammation (Fig. 4C), and an increase in mucus producing goblet cells (Fig. 4D) to a moderate to severe degree as shown in representative digital scans of lung sections (Fig. 4E–H). While pulmonary arterial remodeling, perivascular inflammation, and alveolar inflammation had similar severity scores in the antigen-exposed mice that were transgene-positive or transgene–negative (Fig. 4A–C), the goblet cell score was increased to a significantly lower level in the antigen-exposed transgene-positive mice when compared to the transgene-negative animals (Fig. 4D). Saline exposed groups of transgene-negative or transgene-positive mice showed normal lung histology (Fig. 4A–F).

### Effects of antigen challenge on body and heart weights

**Figure 5:**
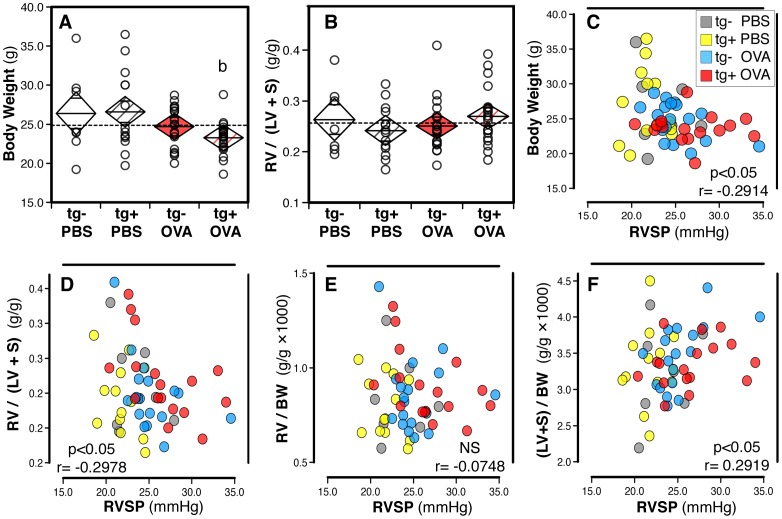
Body and heart weights: effects of the hypomorphic BMPR2 transgene and antigen exposure. (**A, B**) Diamond mean comparison plots show group means, upper and lower 95% confidence limits, overlap marks, for body weight (**A**) and right ventricular weight relative to the weight of the left ventricle and septum, RV/LV+S (**B**). Data are shown for groups of saline (PBS) versus antigen (OVA) exposed mice stratified by transgene-negative (tg−) versus -positive (tg+). The dashed line represents the overall mean. Dots represent data from individual mice. Data were pooled from three independent experiments (n = 7 to 19). Pair-wise comparisons were performed with the Mann-Whitney U test (b: transgene-positive saline vs. antigen, p<0.05). (**C–F**) Dot plots show individual data points for right ventricular systolic pressure (RVSP, mmHg) plotted against body weight (**C**), right ventricular weight relative to the weight of the left ventricle and septum (**D**), right ventricular weight relative to body weight (**E**), and weight of left ventricle and septum relative to body weight (**F**). Data were pooled from three independent experiments (n = 7 to 18 per group). Combined data from saline (PBS) or antigen (OVA) exposed groups of transgene-negative or trangene-positive mice (indicated by color code) were analyzed with the Spearman's Rank Order Correlation Coefficient test (p- and r- values are indicated).

The next analyses were performed to study growth adaptation to antigen exposure, and right heart hypertrophy responses to increased right ventricular systolic pressures (Fig. 5A, B). The antigen exposure protocol slowed growth and this effect was significant in transgene-positive but not transgene-negative mice (Fig. 5A). Consequently, body weight was significantly negatively correlated with right ventricular pressures (Fig. 5C). As expected from previous findings in Dr. West's laboratory with the BMPR2^R899X^ strain [22], right ventricular weights (corrected for the weight of the left ventricle and septum, Fig. 5B, or for body weight) did not increase with antigen exposure, and were not positively correlated with right ventricular systolic pressures (Fig. 5D, E). Surprisingly, the weights of the left ventricle and septum showed a weak positive correlation with right ventricular systolic pressures (Fig. 5F), perhaps reflecting changes in the systemic circulation of antigen-exposed mice.

### Airway inflammation and cellular changes in lung draining lymph nodes

**Figure 6:**
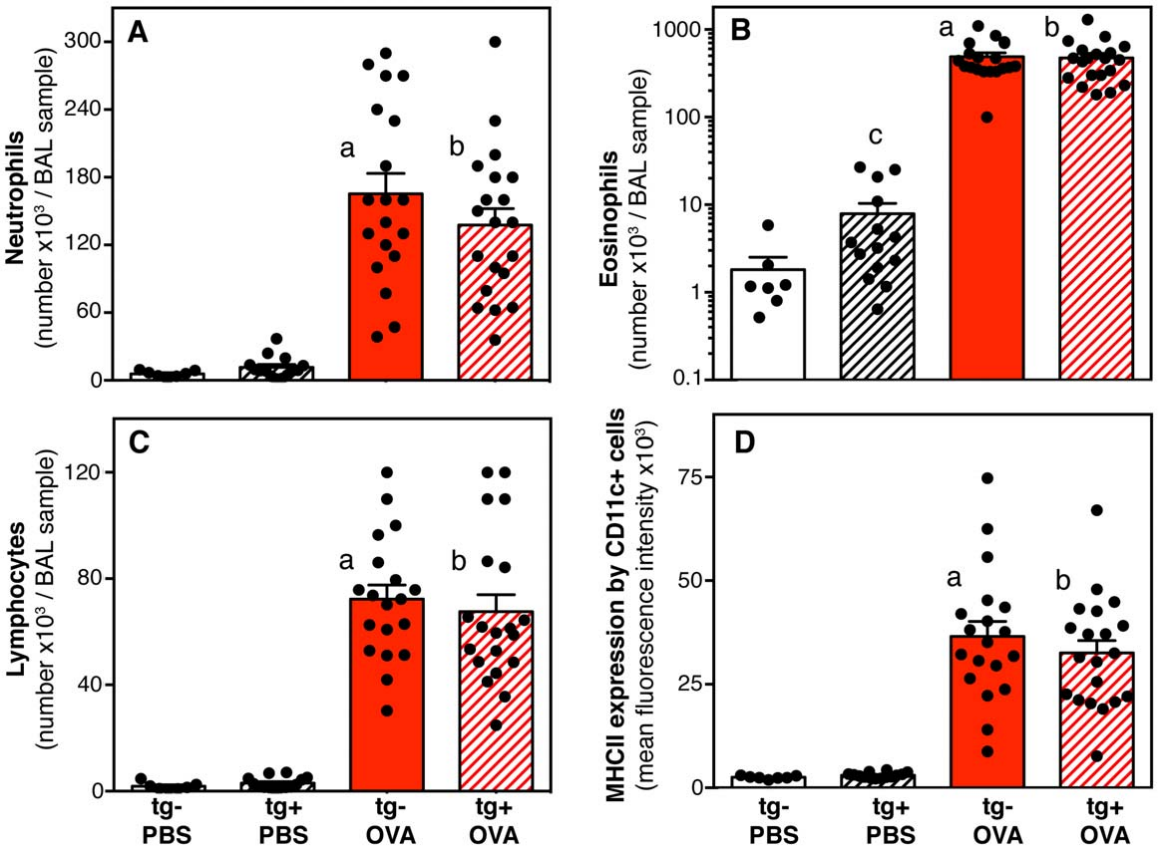
Inflammatory cell responses in the airways: effects of the hypomorphic BMPR2 transgene and antigen exposure. Bar graphs show means and standard error of the means (SEM) for cellular analysis of bronchoalveolar lavage (BAL): numbers per sample of (**A**) neutrophils, (**B**) eosinophils, (**C**) lymphocytes (sum of CD4+ and CD8+ cells) and (**D**) MHCII expression by CD11c+ cells (mean fluorescence intensity, MFI). Data of individual mice are represented as dots. Data were pooled from three independent experiments (n = 7 to 20 per group). Pair-wise comparisons between groups were calculated with the Mann-Whitney U test (p<0.05; a: transgene-negative saline (PBS) vs. antigen (OVA); b: transgene-positive saline vs. antigen; c: saline exposed transgene-negative vs. transgene-positive).

**Figure 7:**
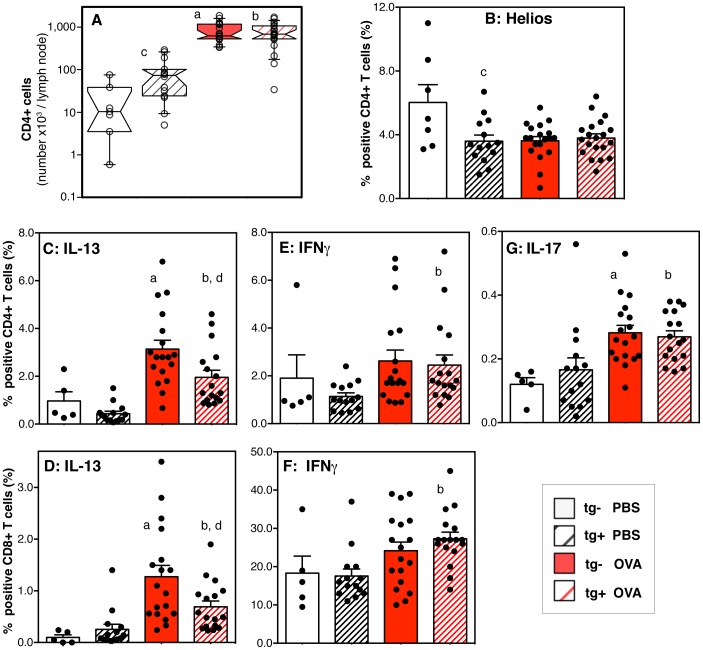
Responses in the lung draining lymph nodes: effects of the hypomorphic BMPR2 transgene and antigen exposure. (**A**) Notched box and whisker plot extending to the 10^th^ and 90^th^ percentiles shows the numbers of CD4+ T cells per lymph node. (**B**) Bar graph shows means and standard error of the means (SEM) of the percentage of CD4+ T cells that were positive for intracellular Helios. (**C–G**) Bar graphs show means and SEM of the percentage of CD4+ or CD8+ T cells that were positive for intracellular cytokines IL-13 (**C, D**), IFNγ (**E, F**), or IL-17A/F (**G**). Data of individual mice are represented as dots. Data were pooled from three independent experiments (n = 6 to 20 per group). Pair-wise comparisons between groups were calculated with the Mann-Whitney U test (p<0.05; a: transgene-negative saline (PBS) vs. antigen (OVA); b: transgene-positive saline vs. antigen; c: saline exposed transgene-negative vs. transgene–positive; d: antigen exposed transgene-negative vs. transgene-positive).

Antigen exposure induced a large influx of neutrophils, eosinophils, and lymphocytes into the airways as measured by analysis of bronchoalveolar lavage (Fig. 6A–C). In addition, bronchoalveolar lavage CD11c+ cells expressed much higher major histocompatibility complex type II (MHCII) levels in antigen-exposed animals when compared to saline exposed (Fig. 6D). Finally, antigen-exposure induced an increase in the numbers of CD4+ T cell in the lung draining lymph nodes, combined with a decrease in the percentage of lymph node CD4+ T cells expressing Helios (Fig. 7A–B). The transcription factor Helios can be expressed by a subtype of T regulatory cells that counterbalance pro-inflammatory effector T cells [31], [32], and in CD4+ T cells differentiating to Th2 or follicular helper cells [33].

The inflammatory responses to antigen exposure were similar in transgene-positive and –negative animals (Fig. 6A–D, 7A–B). In contrast, saline exposed transgene-positive mice had mild inflammation as shown by a significant increase in the numbers of eosinophils in the bronchoalveolar lavage (Fig. 6B), significantly increased numbers of CD4+ T cells (Fig. 7A) and a significantly decreased percentage of Helios+ CD4+ T cells (Fig. 7B) in the lung draining lymph nodes. The inflammatory phenotype in the saline exposed transgene-positive mice was expected based on previous reports from Dr. West's group [22].

### Unique immune phenotype in antigen-exposed BMPR2^R899X^ mice in lung draining lymph nodes

Exposure to antigen induced a significantly increased percentage of CD4+ or CD8+ T cells capable of making IL-13 in both transgen-positive and transgene-negative animals, albeit to a significantly lower level in trangene-positive animals (Fig. 7C, D). In addition, only antigen exposed transgene-positive, but not antigen exposed transgene-negative animals had a significant increase in T cells capable of producing IFNγ that was most noticeable in the CD8+ T cell compartment (Fig. 7E, F). In contrast, the percentage of CD4+ T cells capable of producing IL-17A/F was increased to a similar significant degree in antigen exposed transgene-negative or transgene–positive mice (Fig. 7G).

### Unique cytokine and extracellular matrix protein expression in the right ventricles of transgene-positive mice

**Figure 8:**
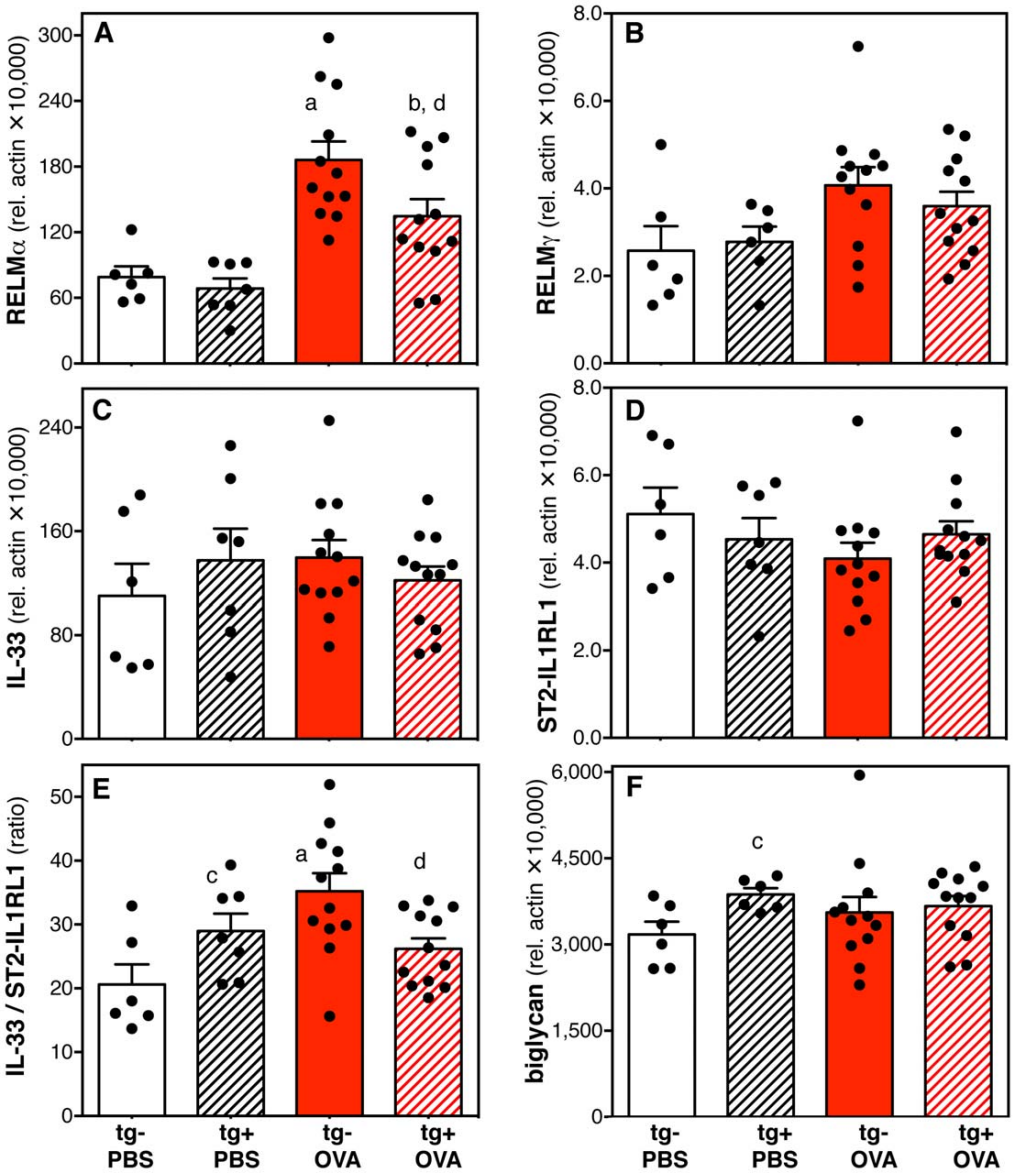
Expression of cytokines and extracellular matrix proteins in the right ventricle: effects of the hypomorphic BMPR2 transgene and antigen exposure. Bar graphs show means and standard error of the means (SEM) of mRNA expression in right ventricles relative (rel.) to β-actin (×10,000) or ratio of expression of cytokine to cytokine receptor. Data are shown for the cytokines RELMα (**A**), RELMγ (**B**), and IL-33 (**C**); the IL-33 receptor ST2-IL1RL1 (**D**); the ratio of IL-33 to ST2-IL1RL1 expression (**E**); and the extracellular matrix protein biglycan (**F**). Data of individual mice are represented as dots. Data were pooled from two independent experiments (n = 6 to 12 per group). Pair-wise comparisons between groups were calculated with the Mann-Whitney U test (p<0.05; a: transgene-negative saline (PBS) vs. antigen (OVA); b: transgene-positive saline vs. antigen; c: saline exposed transgene-negative vs. transgene–positive; d: antigen exposed transgene-negative vs. transgene-positive).

Next, we tested the idea that an immune response in the lungs combined with severe pulmonary vascular remodeling could be reflected in the right ventricle by changes in the expression of cytokines, their receptors and extracellular matrix proteins. The data show that antigen exposure significantly increased the expression of RELMα in the right ventricle in both transgene-negative and –positive mice, albeit to a significantly smaller degree in antigen-exposed transgene-positive animals (Fig. 8A). The expression of RELMγ followed similar trends, but there was no significant difference between groups (Fig. 8B). Transgene-negative antigen-exposed mice showed a non-significant trend towards increase in the expression of IL-33 and decrease of ST2-IL1RL1, an IL-33 receptor, resulting in a significant increase in the ratio of IL-33 to ST2-IL1RL1 when compared to saline controls (Fig. 8 C–E). In contrast, antigen-exposed transgene-positive animals showed no changes in IL-33 or ST2-IL1RL1 expression or in the ratio of IL-33 to ST2-IL1RL1 relative to saline controls (Fig. 8C–E). Furthermore, comparison of the two antigen-exposed groups showed a significantly lower ratio of IL-33 to ST2-IL1RL1 in transgene-positive animals (Fig. 8E). Comparison of the two saline exposed groups revealed significant differences between transgene-negative and transgene-positive mice in the ratio of IL-33 to ST2-IL1RL1 (Fig. 8E) and in the expression of biglycan (Fig. 8F).

## Discussion

Inflammation is now considered to be a critical pathogenetic component in PAH [19], [34]–[39]. Furthermore, recent reports indicate that mutations in the BMPR2 gene and inflammatory responses are linked, together contributing to the pulmonary hypertension phenotype: Dr. Morrell and colleagues, examining smooth muscle cells isolated from patients who carry a BMPR2 mutation found an abnormal inflammatory phenotype in these cells that contributed to the abnormal responses to TGFβ [8]. Dr. Zhang and colleagues showed that monocrotaline induced inflammation was one of the requisite secondary triggers of pulmonary hypertension in mice that have a heterozygous BMPR2 deletion [21].

Our study tested the hypothesis that a Th2 immune response in the lungs induced in response to a mild antigen would trigger or exacerbate the pulmonary hypertension phenotype in mice carrying a BMPR2 transgene [22] that models a hypomorphic mutation found in a family of patients affected with PAH [22], [24]. In our animal facility at New York University, saline exposed BMPR2^R899X^ mice expressing the hypomorphic transgene had mild inflammation and vascular remodeling in the lungs but normal right ventricular systolic pressures relative to transgene-negative littermates. In contrast, antigen-exposed mice that expressed the hypomorphic BMPR2 transgene showed significantly increased right ventricular systolic pressures.

A recent publication by Dr. Le Cras and colleagues [18] tested the same idea using BMPR2 hypomorphic mice that carry one normal BMPR2 allele and one BMPR2 allele that has a deletion of exon 2. The authors found that chronic exposure with house dust mite antigen extract induced the pulmonary hypertension phenotype in wild type and BMPR2 hypomorphic mice to a similar degree, while airway hyperreactivity, a cardinal sign of asthma, was induced to a more severe degree in the BMPR2 hypomorphic animals [18]. The major difference to our experimental system was that exposure to antigen in wild type mice was sufficient to induce the pulmonary hypertension phenotype in the study by Dr. Le Cras and colleagues [18] but not in ours. Compared to house dust mite antigen that primes for Th2 responses when given via the intranasal route [18], the antigen used in our studies, Ovalbumin, is much milder and therefore the mice in our study were primed by the intraperitoneal route with antigen and adjuvants. Ovalbumin is so mild that when given only via the intranasal route to unprimed, naïve animals a tolerance response is induced [28]. In addition, the mouse strains used in our study (FVB) was different from the mouse strain (BALB/c) used by Dr. Le Cras and colleagues [18]. The combined differences in the experimental protocols are likely the reason why antigen-exposure did not induce the pulmonary hypertension phenotype in transgene-negative, wild type animals in our current study, supporting our previously published data [3].

The mechanism that led to the increase in right ventricular systolic pressure in antigen-challenged transgene-positive animals remains unclear. Our data indicate that the severity of pulmonary arterial remodeling or the degree of vessel muscularization in the lungs in response to exposure with the mild antigen were not associated with the increased right ventricular systolic pressures. An alternative explanation, that needs to be addressed in future experiments, would be that the increased right ventricular pressures in the transgene-positive animals reflect a significant difference in the composition of the extracellular matrix of the lungs or heart when compared to the transgene-negative mice, mediated by a skewed balance in BMPR2 and TGFβ receptor signaling in response to injury induced by antigen exposure. The idea that mutations in BMPR2 confer a phenotype of mal-adaption to injury is supported by studies in human patients, which demonstrate a more severe clinical phenotype of PAH in carriers of a mutated BMPR2 allele [40]–[44].

Surprisingly, we report for the first time that the immune response phenotype in the lungs and lung draining lymph nodes corresponded with changes in the right heart based on the measurement of the Th2 response associated genes [45]–[49], RELMα and IL-33, but not the Th2-independent molecules, RELMγ and biglycan. A Th2 inflammatory phenotype in the lungs was reflected by an increase in the ratio of IL-33 expression relative to its receptor ST2-IL1RL1 in the right ventricles and an increase in RELMα expression. This observation was made in two groups of mice: A) Saline-exposed transgene-positive animals that had a mild Th2 type inflammation (mild increase in BAL eosinophils but not neutrophils, mild increase in lung lymph node cellularity). B) Antigen-exposed transgene-negative mice that demonstrated a polarized Th2 response with significantly increased IL-13 but not IFNγ. In a similar pattern, in the right ventricle of antigen-exposed transgene-positive animals the increase in RELMα was less pronounced when compared to antigen-exposed transgene-negative mice and there was no upregulation of the IL-33 to ST2-IL1RL1 expression ratio when compared to saline exposed groups of mice. This may be a reflection of the decreased Th2 polarization in the lymph nodes of these transgene-positive, antigen-exposed mice (significantly less IL-13, increased IFNγ). The decreased Th2 polarization may also explain the development of significantly less pronounced mucus cell hyperplasia in the airways (a sensitive sign of IL-13 dependent Th2 inflammation [30], [50]) of these transgene-positive antigen-challenged animals when compared to transgene-negative antigen-challenged mice. While future studies will need to be designed to elucidate the molecular regulation and functional role of the expression changes of these cytokines in the right heart, our study furthers the emerging topic of the role of inflammation in chronic heart disease [51], [52].

The Th2 response associated cytokines expressed in the right heart could have protective or detrimental effects. For example, IL-33 has been reported to enhance the asthma phenotype induced by exposure to a Th2 antigen [45]–[47], however, by signaling through its receptor, ST2-IL1RL1, IL-33 has been shown to have significant protective function in the left heart [53], [54]. Similarly, RELMα has been shown to enhance hypoxia induced pulmonary hypertension [55], while on the other hand anti-inflammatory effects of this cytokine have been reported [56], [57].

It is tempting to speculate that the changes in the immune response to antigen exposure in mice expressing the hypomorphic BMPR2 transgene were caused by an imbalance of BMPR2 and TGFβ signaling controlling T cell function. T cells are known to express TGFβ receptors and integration of signals from this receptor determines the activity of these cells [58], [59], controlling for example the function of regulatory T cells, the cytokine production profile of effector T cells, and Th17 responses. In addition, signals through BMPR2 have been reported to be important for the development of T cells from thymocytes [60].

In conclusion, our study suggests the possibility that exposure via the airways with mild antigens could exacerbate or trigger the pulmonary hypertension phenotype in patients carrying a BMPR2 mutation, causing an unusual immune response phenotype, and potentially mal-adaptive responses in the right ventricle. Our findings support the idea that there is a molecular connection between a hypomorphic BMPR2, asthma-like responses and pulmonary hypertension as previously suggested by studies performed in pediatric pulmonary hypertension patients [61]. These intriguing findings require further in-depth mechanistic analysis.

## Materials and Methods

### Ethics Statement

All animal experiments were performed according to guidelines outlined by the United States Department of Agriculture and the American Association of Laboratory Animal Care under the supervision and specific approval of the Institutional Animal Care and Use Committees at New York University (IACUC numbers 081114, 111107, most recent approval date: 11/9/2011) (New York, NY). The named IACUC committee specifically approved this study.

### Mice

BMPR2 hypomorphic mice (BMPR2^R899X^) on the FVB background were from Dr. West's laboratory at Vanderbilt University [22]–[24]. This mouse strain was crossed to ROSA promoter transgenic mice, resulting in ROSA26rtTAXTetO(7)-tet-BMPR2(R899X) mice that can express the hypomorphic BMPR2 transgene in every cell type [23], [24]. The BMPR2 transgene has an arginine to nonsense mutation at amino acid 899, modeling a mutation found in a family of patients affected with PAH [22]. Cross-breeding of mice positive or negative for the BMPR2 transgene was performed to generate littermates for the experiments. Only female mice were used for the study being 5–7 weeks of age at the start of the experiment. For each experiment, littermate mice were randomized into cages holding up to 4 mice each. The mice tested negative for mouse pathogens and were housed in filter-top cages under specific pathogen free conditions at Sterling Forest, Tuxedo, NY.

All experiments were performed according to guidelines outlined by the United States Department of Agriculture and the American Association of Laboratory Animal Care under the supervision of the Institutional Animal Care and Use Committee at New York University (New York, NY).

### Antigen priming and challenge

Animals were primed and challenged with antigen as previously published [3]. Briefly, mice were injected intraperitoneally with Ovalbumin (OVA) (grade V; Sigma-Aldrich, St. Louis, MO; 50 µg/dose) adsorbed to Alum (Imject Alum; Thermo Fisher Scientific, Rockford, IL; 2 mg/dose) to prime the immune response at a two-week interval. Two weeks later, the mice were intranasally challenged with OVA (100 µg/dose) for two times each week, for a total of 6 doses given over a three week period. Control mice were given saline (phosphate buffered saline, PBS) intranasally. The experimental conditions are outlined in Figure 1.

### Right Ventricular Systolic Pressure

The analysis was performed without prior knowledge of group designation of the mice [62] using a protocol developed in Dr. West's laboratory [22]. Mice were anaesthetized by intraperitoneal injection of Avertin (tribromoethanol, Sigma-Aldrich, up to 500 mg/kg). Once the pain reflex ceased, mice were shaved with shaving lotion, placed on a surgical table, and fixed with tape. The surgical area was exposed using scissors and surgical forceps. The thyroid grand was dissected upward to expose the right jugular vein. Two sutures were placed under the clearly exposed jugular vein. The cranial suture was tightly tied and the caudal suture was loosely tied. A hole was made between two sutures using small surgical microdissecting scissors. A pressure catheter (F1.4, Millar Instruments, Inc. Houston, Texas) was inserted and advanced into the right ventricle. The pressure signal was monitored to determine the position of the catheter. Once in the correct position, right ventricular pressures were recorded for two minutes. The right ventricular pressure data were analyzed using the LabChart 7 program (ADInstruments, Colorado Springs, CO). Without knowledge of group designation, more than 20 curves were selected randomly for each animal. The difference between maximum and minimum pressure for each curve was measured and averaged to derive the right ventricular systolic pressure for each mouse. Once the catheter was removed, the second suture was tied off tightly. Mice were euthanized with an overdose of sodium pentobarbital.

### Bronchoalveolar lavage and tissue harvest

Bronchoalveolar lavage and tissue harvest was performed as described [3], [27]–[30], [62]. Briefly, a tracheal catheter was inserted and bronchoalveolar lavage (BAL) was performed by gently washing with three 1 ml aliquot of Hanks Balanced Salt solutions. Following BAL, lungs, lung draining lymph nodes and ventricles were recovered. The right lung lobe was snap frozen in liquid nitrogen, the remainder of the lungs was inflated with buffered formaldehyde and removed into formaldehyde for histology. The lung draining lymph nodes were removed for preparation of single cell suspensions. In our laboratory, the BAL does not wash out all inflammatory cells from the lungs, and is performed gently to minimize the potential for mechanical damage of the tissue. This consecutive protocol allows us to reduce the number of animals used for each study.

### Right Ventricular Hypertrophy

The right ventricle, and left ventricle plus septum were removed and weighed. The body weight was also determined. Weight determinations were performed without prior knowledge of group designation of the mice. The data were used to calculate a) the right ventricular weight relative to the combined weight of the left ventricle and septum and b) ventricular weights relative to body weight.

### Quantification of pulmonary vascular muscularization

The degree of muscularization was quantified in sections that were stained by dual-immunohistochemistry with a rabbit anti-von Willebrand factor antibody (Dako, Glostrup, Danemark) recognizing endothelial cells and goat anti-smooth muscle actin antibody (Everest Biotech, Upper Heyford, UK) to detect muscularization as described [25], [26]. Slides were scanned using an Aperio slide scanner and the images analyzed with the VisiomorphDP™ software (Visiopharm, Hoersholm, Denmark). From each slide 60–1000, mean 400, vessels were identified and analyzed for muscularization by calculating a) the length of smooth muscle actin staining relative to the length of the vessel circumference for vessels less than 50 µm, or 50–100 µm in diameter, b) the area of smooth muscle positivity relative to the total area for vessels 50–100 µm in diameter. For each parameter, the vessels were categorized in 3 groups. The categories for the relative length of smooth muscle actin staining were non-muscularized (<0.2); partially (0.2–0.8); fully (>0.8) muscularized. The categories for the percentage of the smooth muscle actin positive area were <10%; 10–30%; >30%.

### Scoring of histological changes

Lung tissue was recovered after taking BAL. The lung was placed into formaldehyde for fixation and preparation of histological slides. Lungs were stained with H&E (hematoxylin and eosin) and PAS (periodic acid schiff). Lung sections were coded and randomized to obscure the group identity. Sections were examined with a light microscope at 200× or 400× magnification. Scores were determined without prior knowledge of group designation of the sections as described [3], [27]–[30]. Random, consecutive view fields were scored. Fifteen to thirty fields per lung were scored, and the mean score was calculated for each parameter. Digital images were taken from slides scanned using the Leica Biosystems SlidePath Gateway and the Leica SCN400 slide scanner (Leica Microsystems Inc., Buffalo Grove, IL).

Pulmonary arterial remodeling was scored on small- to medium-sized arteries that are located close to the airways and could be examined under the view field given with 400× magnification as follows [3]: (1) normal; (2) thickened vascular wall with intact lumen and circular media (all cells follow the form given by the endothelium); (3) the wall is thickened and lined with disorganized layers of cells (cells in the blood vessel wall assume a pattern that differs from the lumen); or (4) thickening is more pronounced as for score ‘3’ and lumen is nearly closed.

Perivascular inflammation was scored as follows [3], [27]–[30]: (1) normal with very few inflammatory cells; (2) scattered inflammatory cells up to two rings in depth; (3) cuffs of inflammatory cells measuring three rings or more in depth; or (4) dense ring of inflammatory cells.

Alveolar inflammation was scored as [3], [27]–[29]: (1) normal; (2) alveolar walls normal, few macrophages in alveoli; (3) mild thickening of alveolar walls and increased alveolar macrophages and eosinophils; or (4) marked thickening of alveolar walls and alveolar multinucleated giant cells, and inflammatory cells (eosinophils, neutrophils, macrophages, lymphocytes) in 30–50% of the field.

Goblet cell hyperplasia [3], [27]–[30]: Medium-sized airways were scored in sections stained with periodic acid Schiff as: (1) less than 5% goblet cells; (2) 5–25%; (3) 25–50%; or (4) more than 50% goblet cells.

### mRNA expression

RNA isolation, cDNA conversion, qPCR and analysis of the data was performed without prior knowledge of group designation of the samples. Total RNA from right heart tissue was isolated with the RNeasy Mini Kit (QIAGEN Inc, Valencia CA) and reverse transcribed using the High-Capacity cDNA Reverse Transcription kit (Applied Biosystems, Foster City, CA). Real time PCR was performed in triplicate with 20 ng of cDNA using the 7900HT Fast Real-Time PCR system (Applied Biosystems). The qPCR for the detection of the expression of the BMPR2 transgene, IL-33, ST2-IL1RL1, biglycan, and β-actin was performed with SYBR Green (Invitrogen, Grand Island, NY). For the specific detection of RELMα and RELMγ, we used the TaqMan Gene expression Assay (Applied Biosystems) based on a FAM labeled probe and the corresponding TaqMan gene expression assay for β-actin. The sequences for the primers or probes, respectively, are indicated in Table 1. The following conditions were used: 95°C for 10 min, followed by 40 cycles of 95°C for 15 s and 60°C for 1 min, followed by a hold at 4°C. Raw data was then analyzed with SDS Relative Quantification Software version 2.3 (Applied Biosystems) to determine cycle threshold (Ct). Mean values were determined and standardized for β-actin. Within each experiment, the β-actin Ct values were tightly clustered with standard deviations of less than 2% of the mean demonstrating the consistent quality of the RNA from each sample (data not shown).

**Table 1:** Sequences of Primers and Probes.

Name	Sequence
BMPR2-transgene-F^1^	GACGAGAGCAACAGGCTGG
BMPR2-transgene-R	CCCTGAAGTTCTCAGCTCTAGATCATTTATC
IL-33-F	ACTGCATGAGACTCCGTTCTG
IL-33-R	CCTAGAATCCCGTGGATAGGC
IL1RL1-ST2-F	TGACTGTCTGGCCCTGAACCT
IL1RL1-ST2-R	TCTCTCCAGAACAGAGCAACCTCAA
Biglycan-F	TGAACCAGGAGCCTTTGATGGC
Biglycan-R	GTCCTCCAACTCAATAGCCTGG
β-actin-F	GGCTGTATTCCCCTCCATCG
β-actin-R	CCAGTTGGTAACAATGCCATGT
RELMα(TaqMan Gene expression assay)	CTTGCCAATCCAGCTAACTATCCCT
RELMγ(TaqMan Gene expression assay)	AAACCTGGCTCATATCCCATTGATG
Actin, β(TaqMan Gene expression assay)	ACTGAGCTGCGTTTTACACCCTTTC

1 Abbreviations: F- forward, R-reverse.

### Immune responses

BAL cells and lung lymph node cells were analyzed by flow cytometry as described [3], [27], [28] using a MACS Quant (Miltenyi Biotec, Auburn, CA) instrument and FloJo (TreeStar Inc, Ashland, OR) software. Cell counts were performed using the feature provided by the MACS Quant flow cytometer. All cell analysis was performed without prior knowledge of the group designation of the samples.

### BAL samples

BAL samples were analyzed as described [27] for the presence of eosinophils (CD11b^high^, CCR3^high^, GR1^low^, CD11c^low-intermediate^, MHCII^low-intermediate^), neutrophils (CD11b^high^, GR1^high^, CD11c^low-intermediate^, MHCII^low-intermediate^), lymphocytes (cells expressing CD4 or CD8 and falling into the lymphocyte gate by forward and side scatter profile), and CD11c+ cells that co-express CD205. The mean fluorescence intensity of CD11c+CD205+ cells stained with anti MHCII antibody was determined as a measure of the capacity of these cells to present antigen [27], [28].

Single cell suspensions from lung draining lymph nodes were analyzed as described [3], [27], [28], [63] following brief fixation with 2% PBS buffered formaldehyde (20–30 minutes at room temperature). The cell suspensions were analyzed for cellularity (cell count per sample) and numbers of T cells (CD3 positive, B220 negative cells that are also positive for CD4 or CD8). CD4+ T cells were intracellularly stained using a published protocol [3], [63] following permeabilization with saponin-containing buffer with anti-Helios antibody and the frequency of this subpopulation was determined.

Intracellular cytokine staining was performed on cell suspensions prepared from the lung draining lymph nodes, as previously described [3], [27]–[29], [63]. The cells were cultured in the presence of phorbol-myristate-acetate (PMA) and ionomycin for 4 h, with the addition of Brefeldin A for the last 2 h of culture. The cells were harvested, fixed in 2% buffered formaldehyde, permeabilized, and stained with anti-cytokine (anti-IL-13, anti–IFN-γ, and anti- IL-17A/F monoclonal antibodies). The cells were surface stained with anti-CD3 combined with anti-CD8α and anti-CD4 labeled monoclonal antibodies. The cells were examined on a MACSQuant cytometer. Electronic gates were set using the forward and side scatter profiles in combination with the surface labels to capture CD3+ T cells that were either CD4+ or CD8+. Intracellular isotype control monoclonal antibodies were used to set the quadrants that demarcated cytokine-positive cells.

The cell populations were analyzed using monoclonal antibodies tagged with Pacific Blue, AlexaFluor, fluorescein (FITC), phycoerythrin (PE), peridinin-chlorophyll (PerCP), Allophycocyanin (APC), or cyanine (Cy) tandem dyes that were purchased from BD Bioscience (San Jose, California), Ebioscience (San Diego, California) or Biolegend (San Diego, California). BAL cells were stained with anti-CD11c-PacificBlue (clone N418); anti-CD11b- PE (clone M1/70); anti-MHCII-FITC (I-A/I-E, clone M5/114.15.2); anti-Ly-6G/Ly-6C-APC-Cy7 (clone GR1); PerCp-anti-CD4 (clone GK1.5) and PerCP-anti-CD8α (clone 53-6.7); anti-CCR3-AlexaFluor 647 (clone TG14, Biolegend, or clone 83103, BD Bioscience); anti-CD205-PE-Cy7 (clone Dec205 Biolegend). Lung lymph node cells were stained with anti-CD45R/B220-PacificBlue (clone RA3-6B2, Biolegend); anti-CD3-FITC (clone 17A2, ebioscience), anti-CD8α-PE-Cy7 (clone 53-6.7, Biolegend), anti-CD4-APC-Cy7 or -PerCp (clone GK1.5, Biolegend), anti-Helios-APC (clone 22F6, Biolegend). Intracellular staining was performed with anti-IL-13-PE (clone eBio13A, Ebioscience), anti-IFNγ –AlexaFluor 645 (clone XMG1.2, Biolegend), and anti-IL-17A/F-PE-Cy7 (clone TC11-18H10.1, Biolegend), on cells that were surface stained with anti-CD3-FITC, anti-CD8α-PerCP, and anti-CD4-APC-Cy7. IgG1-isotype control antibodies were from ebioscience or biolegend.

### Statistical analysis

Statistical analysis and graphs were generated using the Aable 3 software (Gigawiz) or Prism 5 (Graphpad). Data from multiple groups were analyzed for significant differences using the Kruskal Wallis test (tie corrected). Pair-wise comparisons were conducted with the unpaired, two-tailed Mann-Whitney U test, or the unpaired, two-tailed t test with Welch's correction for unequal variances. Categorical data were analyzed with the chi-square test. Analysis for correlation was performed with the Spearman's Rank Order Correlation Coefficient test (two-tailed). A p value <0.05 was considered to mean statistical significance.
